# 

**Published:** 1900-03

**Authors:** 


### THE BRISTOL
### Medico-Chirurgical Journal.

A JOURNAL OF THE MEDICAL SCIENCES FOR THE WEST OF ENGLAND AND SOUTH WALES

*PUBLISHED UNDER THE AUSPICES OF*

*THE BRISTOL MEDICO-CHIRURGICAL SOCIETY.*

21

EDITOR:

R. SHINGLETON SMITH, M.D., B.Sc.,

WITH WHOM ARE ASSOCIATED

J. MICHELL CLARKE, M.A., M.D., J. LACY FIRTH, M.S., M.D., AND JAMES SWAIN, M.S., M.D.

ASSISTANT-EDITOR:

P. WATSON WILLIAMS, M.D.

EDITORIAL SECRETARY:

JAMES TAYLOR.

"Scire est nescire, nisi id me

Scire alius sciret."

LIBRARY [illegible]
JUN -3 1901
174062.

*VOL. XVIII.*

BRISTOL: J. W. ARROWSMITH.
LONDON: J. & A. CHURCHILL, 7 GREAT MARLBOROUGH STREET.
1900.

WI
BR323

J.W. Arrowsmith Publisher Bristol

### CONTENTS OF VOLUME XVIII.

*Page*

Notes on Some Thermal, Hydro-Thermal, Electric and Hydro-Electric Procedures, and the Indications for their Use. By C. J. WHITBY, B.A., M.D. Cantab. ... ... ... ... ... ... ... ... ... ... ... 1

Rheumatic Disease of the Cardiac Muscle. By THEODORE FISHER, M.D. Lond., M.R.C.P. ... ... ... ... ... ... ... ... .. ... 16

Vascular Caruncle of the Urethra. By A. E. AUST LAWRENCE, M.D. 23

Non-Bacillary Croup. By J. O. SYMES, M.D., D.P.H. Lond. ... ... 25

The Spontaneous Disappearance of a Sarcomatous Tumour. By G. MUNRO SMITH, M.R.C.S. ... ... ... ... ... ... ... ... .. 30

Presentation to Mr. L. M. Griffiths ... ... ... ... ... ... ... ... 97

A Retrospect of a Second Series of Fifty Consecutive Intra-Abdominal Operations. By JAMES SWAIN, M.S., M.D. Lond., F.R.C.S. Eng. ... ... ... ... ... ... ... ... ... ... ... ... 103

Cases of Acute Intestinal Obstruction Treated by Operation. By W. H. HARSANT, F.R.C.S. ... ... ... ... ... ... ... ... ... 118

Abdominal Hysterectomy for Fibroids of the Uterus, with Retro-Peritoneal Treatment of the Stump, and Notes of two Cases. By W. C. SWAYNE, M.D. Lond.... ... ... ... ... ... ... ... ... 123

Two Cases of the Sporadic Form of Epidemic Cerebro-Spinal Meningitis. By J. MICHELL CLARKE, M.A., M.D. Cantab., F.R.C.P. Lond. ... ... ... ... ... ... ... ... ... ... ... 128

Swedish Medical Gymnastics in Chronic Diseases of the Heart. By E. CHRISTOFFERSON ... ... ... ... ... ... ... ... ... ... 134

Note on a Pre-Exanthematous Sign of Measles. By P. WATSON WILLIAMS, M.D. Lond.... ... ... ... ... ... ... ... ... ... ... 139

Reminiscences of the Bristol Royal Infirmary. By ARTHUR W. PRICHARD, M.R.C.S. ... ... ... ... ... ... ... ... ... 193

Abdominal Incisions. By THOMAS CARWARDINE, M.S. Lond., F.R.C.S. 204

CONTENTS.

Page

Spindle-celled Saracoma of the Hard Palate. By W. J. GREER, F.R.C.S. Irel. ... ... ... ... ... ... ... ... ... ... ... ... 211

The X-Ray History of a Fracture. By JAMES TAYLOR, M.R.C.S. ... 214

One Thousand Consecutive Inductions of General Anæsthesia. By C. HAMILTON WHITEFORD, M.R.C.S., L.R.C.P. ... ... ... ... 217

The Inebriates' Acts, 1879—1899. By WILLIAM COTTON, M.A., M.D. Edin., D.P.H. ... ... ... ... ... ... ... ... ... ... ... ... 222

The Dietetic Treatment of Zymotic Enteritis, especially with regard to the use of Raw Meat Juice. By F. PERCY ELLIOTT, M.B., C.M. Aberd. ... ... ... ... ... ... ... ... ... ... ... ... 225

Some Modern Aspects of Preventive Medicine. By DAVID SAMUEL DAVIES, M.D. Lond., D.P.H., President ... ... ... ... ... ... 289

A Plea for Sanatoria for the Poorer Consumptives. By LIONEL A. WEATHERLY, M.D. ... ... ... ... ... ... ... ... ... ... ... ... 305

Three Abdominal Cases Presenting Some Unusual Features. By A. W. PRICHARD, M.R.C.S. ... ... ... ... ... ... ... ... ... 313

A Case of Diffuse Leontiasis Ossea. By E. H. EDWARDS STACK, M.B., F.R.C.S. ... ... ... ... ... ... ... ... ... ... ... ... 316

Forty Cases of Operation for Appendicitis. By CHARLES A. MORTON, F.R.C.S. ... ... ... ... ... ... ... ... ... ... ... ... ... ... 320

Five Cases of Pelvic Disease Treated by Laparotomy. By ERNEST W. HEY GROVES, M.B., B.Sc. (Lond.) ... ... ... ... ... ... ... 330

The Technique of Halsted's Operation for Cancer of the Breast. By C. HAMILTON WHITEFORD, M.R.C.S., L.R.C.P. ... ... ... 336

PROGRESS OF THE MEDICAL SCIENCES ... ... ... 33, 141, 229, 338

REVIEWS OF BOOKS ... ... ... ... ... ... ... ... 45, 155, 242, 353

NOTES ON PREPARATIONS FOR THE SICK ... ... ... 72, 173, 275, 361

MEETINGS OF SOCIETIES ... ... ... ... ... ... ... ... 75, 175, 381

THE LIBRARY OF THE BRISTOL MEDICO-CHIRURGICAL SOCIETY ... ... ... ... ... ... ... ... ... ... ... 85, 182, 277, 385

OBITUARY ... ... ... ... ... ... ... ... ... ... ... ... ... ... ... 185, 390

LOCAL MEDICAL NOTES ... ... ... ... ... ... 88, 186, 282, 387

SCRAPS ... ... ... ... ... ... ... ... ... ... ... 95, 191, 287, 391

### The Library of the Bristol Medico-Chirurgical Society.

*The following donations have been received since the publication of the List in December:*

*February 28th, 1900.*

L. M. Griffiths (1) ... ... ... ... ... ... ... 1 volume.

Manchester Medical Society (2) ... ... ... ... 4 volumes.

Mason University College (3) ... ... ... ... 2 ,,

Pathological Society of London (4) ... ... ... 1 volume.
Victor G. Plarr (5) ... ... ... ... ... ... ... ... 1 „
Royal Academy of Medicine in Ireland (6) ... 1 „
J. Taylor ... ... ... ... ... ... ... ... ... 1 „

Unbound periodicals have been received from the Manchester Medical Society.

---

### THIRTY-FIFTH LIST OF BOOKS.

The titles of books mentioned in previous lists are not repeated.

The figures in brackets refer to the figures after the names of the donors, and show by whom the volumes were presented. The books to which no such figures are attached have either been bought from the Library Fund or received through the *Journal*.

**Adami and C. F. Martin, J. G.** *Report on Observations made upon the Cattle at the Experimental Station at Outremont, P.Q.* 1899
**Allbutt, T. C.** [Ed.] *A System of Medicine.* Vol. VIII. ... ... ... ... 1899
**Barwise, S.** ... *Practical Hints on the Analysis of Water and Sewage* ... 1899
**Bell, A. E.** ... ... *The Pasteurisation and Sterilisation of Milk* ... ... ... 1899
**Broadbent, Sir W. H. and J. F. H.** *Heart Disease* ... ... ... 3rd Ed. 1900
**Bruce, J. M.** ... *The Principles of Treatment* ... ... ... ... ... ... ... 1899
**Burghard, W. W. Cheyne and F. F.** *A Manual of Surgical Treatment.* Part II. ... ... ... ... ... ... ... ... ... ... 1899
*Catalogue of the Library of the Ophthalmological Society of the United Kingdom* ... ... ... ... ... ... ... 3rd Ed. 1899
**Cheyne and F. F. Burghard, W. W.** *A Manual of Surgical Treatment.* Part II. ... ... ... ... ... ... ... ... ... ... 1899
**Davis, A. E.** ... *The Refraction of the Eye* ... ... ... ... ... ... ... 1900
**Foster, M.** ... ... *Claude Bernard* ... ... ... ... ... ... ... ... ... ... 1899
**Foxwell, A.** ... *The Causation of Functional Heart Murmurs* ... ... 1899
**Griffiths, W. H.** *Lessons on Prescriptions and Prescribing* [3rd Ed.] 1899
**Hall and J. A. Menzies, I. W.** *Golden Rules of Physiology* ... ... ... [1900]
**Harding, W.** .. *Mental Nursing* ... ... ... ... ... ... ... ... ... ... 1899
**Hill, L.** ... ... *Manual of Human Physiology* ... ... ... ... ... 1899
**Hime, M. C.** ... *Schoolboys' Special Immorality* ... ... ... 2nd Ed. 1899
**Hinshelwood, J.** *Letter-, Word- and Mind-Blindness* ... ... ... ... 1900
**Jardine, R.** ... *Lectures on Hæmorrhage and Eclampsia* ... ... ... ... 1899
„ ... *Text-Book of Midwifery* ... ... ... ... ... ... ... ... 1899
**Lawrie, J. M.** ... *Fifty Consecutive Cœliotomies* ... ... ... ... ... ... 1899
**Lehfeldt, R. A.** ... *A Text-Book of Physical Chemistry* ... ... ... ... [1899]
**Leser, E.** ... ... *Operations-vademecum* ... ... ... ... ... ... ... ... 1900
**Linn, T.** ... ... *The Health-Resorts of Europe* ... ... ... ... ... ... 1899
**Luff and F. J. M. Page, A. P.** *A Manual of Chemistry* ... [2nd Ed.] 1900
**M'Kendrick, J. G.** *Hermann Ludwig Ferdinand von Helmholtz* ... ... ... 1899
**Martin, J. G. Adami and C. F.** *Report on Observations made upon the Cattle at the Experimental Station at Outremont, P.Q.* 1899
**Menzies, I. W. Hall and J. A.** *Golden Rules of Physiology* ... ... ... [1900]
*Merck's Manual of the Materia Medica* ... ... ... ... ... ... ... ... .. 1899
**Moure, E. J.** ... *Traitement des Tumeurs malignes des Fosses nasales* ... 1899

**Murrell, W.** ... *Aids to Materia Medica.* 3 parts. ... ... ... 1898–1900
**Norris and C. A. Oliver, W. F.** [Eds.] *System of Diseases of the Eye.* Vol. IV. ... ... ... ... ... ... ... ... ... 1900
**Oliver, W. F. Norris and C. A.** [Eds.] *System of Diseases of the Eye.* Vol. IV. ... ... ... ... ... ... ... ... ... 1900
**Page, A. P. Luff and F. J. M.** *A Manual of Chemistry* ... [2nd Ed.] 1900
**Plarr, V. G.** ... *List of Lecturers and Lectures at the Royal College of Surgeons of England, 1810—1900* ... ... ... (5) 1900
**Power, D'A.** ... *How Surgery became a Profession in London* ... ... ... 1899
**Pye, [W.]** ... ... *Surgical Handicraft.* 4th Ed. (Ed. by B. M. H. Rogers) 1900
**Richardson, Sir B. W.** *Vita Medica* ... ... ... ... ... ... ... ... ... 1897
**Shuttleworth, G. E.** *Mentally-Deficient Children* ... ... ... ... 2nd Ed. 1900
**Stonham, C.** ... *A Manual of Surgery.* Vol. III. ... ... ... ... ... 1899
**Wilson, G. R.** ... *Vice and Insanity* ... ... ... ... ... ... ... ... 1899
**Yearsley, M.** ... *Nursing in Diseases of the Throat, Nose, and Ear* ... 1899

### TRANSACTIONS, REPORTS, JOURNALS, &c.

American Journal of Medical Sciences, The ... ... Vol. CXVIII. 1899
American Ophthalmological Society, Transactions of the Vol. VIII., Part 3 1899
American Surgical Association, Transactions of the ... Vol. XVII. 1899
Annual and Analytical Cyclopædia of Practical Medicine ... Vol. IV. 1899
Archives de Neurologie ... ... ... ... 2e Sér., Tomes VII., VIII. 1899
Archivio di Ortopedia ... ... ... ... ... .. Vols. XIV., XV. 1897–98
Association of American Physicians, Transactions of the Vol. XIV. 1899
Birmingham Medical Review, The ... ... ... Vols. XLV., XLVI. 1899
Bristol Health Report, 1898 ... ... ... ... ... ... ... ... (1) 1899
British Almanac, The ... ... ... ... ... ... ... ... ... ... ... 1900
British Medical Journal, The .. ... ... ... ... ... Vol. II. for 1899
Buffalo Medical Journal ... ... ... ... ... ... ... Vol. XXXVIII. 1899
Bulletin de l'Institut international de Bibliographie ... ... ... ... 1898
Centralblatt für Chirurgie ... ... ... ... ... ... ... ... ... ... 1899
Cincinnati Lancet-Clinic, The ... ... ... ... ... Vol. LXXXII. 1899
Clinical Journal, The ... ... ... ... ... ... Vols. XII.—XIV. 1898-99
Clinical Society's Transactions ... ... ... ... ... ... Vol. XXXII. 1899
Deutsche medicinische Wochenschrift ... ... ... ... ... ... (2) 1888
Dublin Journal of Medical Science, The ... ... ... ... Vol. CVIII. 1899
Edinburgh Medical Journal, The ... ... ... ... N.S., Vol. VI. 1899
Edinburgh Obstetrical Transactions ... ... Vols. XI.—XVIII. 1886-93
Gazette des Hôpitaux ... ... ... ... ... ... ... ... ... ... 1898
Glasgow Medical Journal, The ... ... ... Vols. (2) IX., 1877; LII. 1899
Hazell's Annual ... ... ... ... ... ... ... ... ... ... ... 1900
Hospital, The ... ... ... ... ... ... ... ... ... ... Vol. XXVI., 1899
Hospitals—
Glasgow Hospital Reports ... ... ... ... ... ... ... ... Vol. II. 1900
Saint Bartholomew's Hospital Reports ... ... ... ... Vol. XXXV. 1900
Internationale Oogheelkundig Congres Te Utrecht, Verslag der Algemeene Zittingen van het Negende ... ... ... ... ... ... 1899
Iowa State Medical Society, Transactions of the ... ... Vol. XVII. 1899
Jahresbericht über die Leistungen und Fortschritte in der Gesammten Medicin ... ... ... ... ... ... ... ... ... (2) 1886, Bd. II. 1887

Jenner Institute of Preventive Medicine, Transactions of the 2nd Ser. 1899

Journal of Laryngology, Rhinology, and Otology ... ... Vol. XIV. 1899

Journal of the American Medical Association, The ... Vol. XXXIII. 1899

Journal of the Boston Society of Medical Sciences ... ... Vol. III. 1898-99

Lancet, The ... ... ... ... ... ... ... ... ... ... ... Vol. II. for 1899

Laryngological Society of London, Proceedings of the ... ... Vol. VI. 1899

London Medical and Surgical Journal, The... ... ... (3) Vol. VIII. 1836

Medical Chronicle, The ... ... ... ... ... ... ... ... 3rd Ser., Vol. I. 1899

Medical Directory, The ... ... ... ... ... ... ... ... ... ... ... ... 1900

Medical News, The ... ... ... ... ... ... ... ... ... ... Vol. LXXV. 1899

Medical Press and Circular, The ... ... ... ... ... ... [Vol. CXIX.] 1899

Medical Record ... ... ... ... ... ... ... ... ... ... ... ... Vol. LVI. 1899

Medical Review, The ... ... ... ... ... ... ... ... ... ... Vol. II. 1899

New Hampshire Medical Society, Transactions of the ... ... ... ... 1899

Newspaper Press Directory ... ... ... .. ... ... ... ... ... ... 1900

New York Medical Journal, The ... ... ... ... ... ... ... Vol. LXX. 1899

New York, Transactions of the Medical Society of the State of ... (2) 1865

Odontological Society of London, Transactions of the ... Vol. XXXI. 1899

Ophthalmological Society of the United Kingdom, Transactions of the Vol. XIX. 1899

Pathological Society of London, Transactions of the ... (4) Vol. L. 1899

Philadelphia Medical Journal, The ... ... ... ... ... ... Vol. IV. 1899

Practitioner, The ... ... ... ... ... ... ... ... ... ... ... Vol. LXIII. 1899

Public Health ... ... ... ... ... ... ... ... ... ... ... ... Vol. XI. 1898-99

Quarterly Medical Journal, The ... ... ... ... ... ... ... Vol. VII. 1898-99

Retrospect of Medicine, Braithwaite's ... ... ... ... Vol. CXX. 1900

Revue hebdomadaire de Laryngologie [etc.] ... ... ... Tome XIX. 1899

Royal Academy of Medicine in Ireland, Transactions of the (6) Vol. XVII. 1899

Sanitary Record, The ... ... ... ... ... ... ... ... ... Vol. XXIV. 1899

Scottish Medical and Surgical Journal, The ... ... ... ... Vol. V. 1899

Smithsonian Institution, Annual Report of the, for 1896, 1897 ... ... 1898

University Medical Magazine ... ... ... ... ... ... ... ... Vol. XI. 1899

West London Medical Journal ... ... ... ... ... ... ... ... Vol. IV. 1899

Whitaker's Almanack ... ... ... ... ... ... ... ... ... ... ... ... ... 1900

Who's Who ... ... ... ... ... ... ... ... ... ... ... ... ... ... ... 1900

Year-Book of Scientific and Learned Societies ... ... ... ... ... (3) 1885

### Local Medical Notes.

### BRISTOL.

"JOURNAL" COMMITTEE.—Our readers will have observed some changes on our title page. The Assistant Editor, Mr. L. M. Griffiths, after 17 years of unremitting devotion to the *Journal*, has resigned his office, and the Committee of the Society have reluctantly accepted his resignation. Some steps have been taken to convey to Mr. Griffiths, in a substantial and suitable form, an expression of the widely-spread gratitude which a large proportion of the members of the Society and the readers of the *Journal* feel to Mr. Griffiths for his great services to the profession for many years. Further details of this will shortly be announced: meanwhile, the Editor, who is Treasurer of the Fund, will be glad to receive any further contributions.

Mr. W. H. Harsant, a member of the Journal Committee for nearly ten years, has also retired, and the Committee has conveyed to Mr. Harsant their thanks for his extremely valuable services during the long period of his connection with the *Journal*.

Dr. Bertram M. H. Rogers has also resigned his position on the Journal Committee, and in accepting his resignation the Committee desire to express their cordial appreciation of the large amount of necessary and valuable work he has done during a period of five years.

New appointments have been made by the Committee of the Society as follows:—

Dr. P. Watson Williams is to succeed Mr. Griffiths in the office of Assistant Editor, and we have no doubt that his experience in similar work, and his energy in doing it, will be of great service to the *Journal*.

The other vacancy on the Committee has been filled by the appointment of Dr. J. Lacy Firth, who, as Assistant Surgeon to the Bristol General Hospital, will be able to give the Committee much assistance on surgical questions.

Princess Christian Hospital.—The staff of this hospital having been selected so largely from Bristol, and the connection of the donor with this city, gives the subject an especial local interest, and hence we have no hesitation in giving some details on the subject. The donor and his staff are now on their way to the destination which may be allotted them by the War Office, and we trust that this hospital is destined to be of great service to our soldiers in South Africa.

The "Princess Christian Hospital" has been presented to the Government by Mr. Alfred Mosely, of West Lodge, Hadley Wood, Middlesex. This munificent gift has been entirely equipped and will be supported by the generous donor, who accompanies it to the Cape, and whose knowledge of the country and climate, gained through twenty-five years' connection with South Africa, will prove of considerable value to the efficient working of the hospital. No trouble or expense has been spared to make the hospital in every way worthy of the patronage of Her Royal Highness Princess Christian, who has graciously consented for it to bear her name.

Major H. B. Mathias, D.S.O., R.A.M. Corps. who is a brother of the gallant Colonel H. H. Mathias, V.C., etc., of Dargai fame, goes out as Government Representative with the hospital.

The civil staff will consist of the following:—

*Chief Surgeon.*—J. Paul Bush, M.R.C.S. Eng., Surgeon to the Bristol Royal Infirmary; Lecturer on Operative Surgery, University College, Bristol; Chief Surgeon, Bristol Constabulary.

*Medical Officers.*—G. V. Worthington, M.A., M.B., B.C. Cantab., M.R.C.S. Eng., late Senior House-Surgeon, St. Bartholomew's Hospital, London; late District Medical and Sanitary Officer, S. Canara, India. E. A. Nathan, M.D., B.S. London, M.R.C.S. Eng., late House-Surgeon and House-Physician, St. Mary's Hospital, London. A. L. Flemming, M.R.C.S. Eng., late Resident Casualty Officer, Bristol Royal Infirmary.

*Dressers.*—A. B. Cridland, M.R.C.S. Eng., House-Surgeon, Bristol Eye Hospital; late Casualty Officer, Bristol Royal Infirmary. E. M. Pearse, M.R.C.S. Eng., late Casualty Officer, Bristol Royal Infirmary.

*Nursing Staff.*—Sister E. C. Laurence, of Guy's Hospital, who will be the Sister-in-charge, and Sisters M. Leng, E. Atkins, E. M. Fisher, S. A. Snell, and F. Baker.

There will also be six non-commissioned officers and twenty-six hospital orderlies.

The building will consist of four pavilions, each 130 feet long, and containing 25 beds—100 in all, with surgery, operation room, nurses' room, fitted bath-rooms, washing room for the men, and lavatories attached.

The structure will be of corrugated iron, hung inside with greenish-tinted canvas. The wards will be comfortably furnished with bedsteads fitted with spring mattresses of the newest design, folding washstands, invalid tables and chairs, and every little detail likely to make them more comfortable and home-like. The surgeries and operation room will be fitted with all the latest improvements, including a very complete Röntgen Ray apparatus, with all the latest inventions for localising foreign bodies, etc. The accessories include the new mercurial interrupter for making and breaking the current, and driven by a special motor of its own. This interrupter gives a particularly steady and distinct image with the fluorescent screen, and it also has the advantage of diminishing the length of time-exposure. An expert in this department is included in the list of medical officers.

In addition to the main buildings, there will be three stores for the warehousing of the ample supply of provisions, invalid specialities, stimulants, drugs, etc. There will also be a separate building for the storage of the linen, a laundry, and a very complete kitchen of considerable size. In charge of the culinary department will be a professed cook and assistants. Another large structure will contain a central mess-room, and sleeping accommodation for the doctors, nurses, and other members of the Staff, who will number in all about fifty persons.

The plans and specifications of the hospital were prepared by Mr. Fred. W. Marks, A.R.I.B.A., of 3 Staple Inn, W.C., and the work has been admirably carried out under his superintendence by Messrs. Humphreys, Limited, of Knightsbridge.

Public Health.—The Barton Regis Rural District Council have made arrangements by which medical men in the district can obtain, free of charge, bacteriological cultures in cases of diphtheria, and have blood examined by Widal's test in suspected cases of enteric fever. Culture outfits are kept at depôts in various parts of the district.

Health Report.—Dr. D. S. Davies reports that 9,345 births and 5,840 deaths were registered during 1899. The births correspond to a rate of 29.2 per 1000 persons living, as compared with 28.7 in 1898, and the death-rate was 18.2 per 1000, against 17.2 per 1000 in 1898. The deaths of infants last year numbered 1,473 and were in the proportion of 158 per 1000 births registered, against 164 per 1000 in 1898. The zymotic death-rate was 1.9 per 1000 living, against 2.7 in the previous year. The deaths due to the zymotic diseases were 595, of which 41 were due to measles, 13 to scarlet fever, 33 to diphtheria, 119 to whooping-cough, and 355 to diarrhœa.

Mr. Colston Wintle has been elected vice-chairman of the Health Committee of the Bristol Town Council.

University College, Bristol.—Mr. P. G. Stock, M.R.C.S., L.R.C.P., and Mr. F. W. Cotton, M.R.C.S., L.R.C.P., have joined the Royal Army Medical Corps; the former appointment being made by the War Office on the nomination of the Medical Faculty of the University College, Bristol; the latter gained the fifth position in the competitive entrance examination.

Royal Infirmary.—At a special meeting of the committee, held on November 28th, it was decided to sell sufficient capital stock to provide £8,000 to be applied towards the cost of the Nurses' Home and laundry. The committee of this institution recently offered to make 24 beds available for soldiers of the Gloucestershire Regiment invalided from South Africa and requiring active medical or surgical treatment. The War Office has accepted the proposal.

General Hospital.—Dr. A. J. Harrison has been re-appointed Honorary Physician. Dr. J. Odery Symes has been appointed Assistant Physician.

Royal Hospital for Sick Children and Women.—Mr. H. S. Jenkins has been appointed House Surgeon *vice* Dr. W. Milligan, resigned.

Dispensary.—The annual report of the committee has just been issued, and shows that during 1899 there were 10,835 patients treated, who, with 1,165 remaining at the end of 1898, made a total of 12,000. During 1898 there were 10,176 patients treated. The report adds that during the past year medical assistance has been given in 25 difficult midwifery cases; and that the three beds in the Convalescent Home are much appreciated by the patients.

Royal Victoria Jubilee Convalescent Home.—At a meeting of the committee the Society of Merchant Venturers made an application for 12 beds to be reserved for convalescent sick or wounded soldiers returning from South Africa. The expenses in connection with these beds will be defrayed by the Society. Private H. Edmonds, of the 3rd Grenadier Guards, is the first patient to be received.

Royal Victoria Homes for Inebriates.—The Home Secretary, on the application of the Manager, has extended the certificate to the Brentry Inebriate Reformatory for Females to allow of the reception of 40 females over and above the 35 authorised by the original certificate. He has also certified as an inebriate reformatory for males, under the Inebriates Act, 1898, certain lands and buildings situate at Brentry.

At a meeting of the Newport (Mon.) Corporation, held on January 9th, it was decided to contribute £1,000 to the funds of the Royal Victoria Homes, Brentry, in consideration of having seven beds reserved for a period of 25 years.

### BATH.

Royal United Hospital.—The annual meeting of this institution was held on January 29th, under the presidency of the Mayor. The report stated that 1,287 in-patients had been admitted, and that 8,941 out-patients had been treated, being an increase in each case as compared with 1898. The financial statement showed that the income amounted to £7,421 and the expenditure to £7,768. The report alluded to the services rendered to the hospital by the late Mr. H. Culliford Hopkins, first as house surgeon, and then, since 1876, as registrar and curator of the museum, and added that his name would be handed down as one of the most zealous of workers for the hospital.

Eye Infirmary.—The annual meeting of this institution was held on February 16th. The medical report stated that 268 in-patients and 1,749 out-patients had been treated. The financial statement showed that there remained a favourable balance of £31. On the motion of the Rev. B. N. Thompson, it was decided that an honorarium be awarded to Mr. W. Beaumont, the honorary surgeon to the Infirmary.

Eastern Dispensary.—Mr. R. A. Bayliss has been appointed Honorary Medical Officer *vice* Dr. J. M. Harper, resigned.

Western Dispensary.—The annual meeting of this institution was held on January 26th. The medical report showed that 932 cases had been treated during 1899. The financial statement showed a favourable balance of £102. On the proposition of the chairman, a resolution was unanimously passed expressing regret at the loss the dispensary had sustained by the death of Mr. H. Culliford Hopkins, who for 28 years had been one of the medical officers, and the committee added that the poor would long remember him as their friend and kind benefactor. Mr. G. E. Bloxham has been appointed Medical Officer *vice* Mr. H. C. Hopkins, deceased.

### CARDIFF.

Home for Inebriates.—A sub-committee of the Watch Committee has been considering what course should be taken in connection with the Inebriates Act. Dr. Braithwaite from the Home Office met the sub-committee, when the question of erecting a home at Cardiff for 50 inmates (25 males and an equal number of females) was fully discussed. They recommended that 10 acres of land should be purchased, and that a home be erected at a cost of £10,000. The cost of maintenance was estimated at 15s. per patient per week, towards which the government allow half-a-guinea, and it was considered that the annual expenditure would be about £830. The Committee unanimously adopted the report. It is expected that such an institution will be sufficient to accommodate all those who come under the provisions of the Act in South Wales and Monmouthshire.

Public Health.—Dr. W. G. Savage has been appointed Bacteriologist and Lecturer at the Cardiff and County Public Health Laboratory.

Infirmary.—At the meeting of the committee, held on November 8th, 1899, the financial position of the institution was considered. The Chairman of the Finance Committee stated that by the end of the year the adverse balance would be £7,000, and added that they were at present spending £9,000 per annum, whilst the receipts were only £7,000. At a meeting of the committee, held on November 15th, 1899, it was decided that a women's ward should be closed on November 30th. It is estimated that by closing the ward about £1,172 will be saved annually, but it is hoped that increased subscriptions will permit of the re-opening of the ward at an early date.

At a special meeting of the Executive Committee of the Cardiff Infirmary, held on January 19th, the new rules which have been recommended by a sub-committee were considered. It was resolved: "That no member of the honorary medical staff shall hold office after attaining the age of 60 years. No physician, surgeon, ophthalmic surgeon, gynæcologist, or dental surgeon shall hold office for a longer period than 20 years."

Seamen's Hospital.—A very successful bazaar was held in Cardiff recently, in aid of the fund for the new seamen's hospital. At the opening of the bazaar Mr. D. Thomas, M.P., stated that he had received £1,000 from an anonymous donor towards the institution, and added that the King of Italy had sent a very beautiful specimen of mosaic work. The net proceeds of the bazaar realised £4,276.

At a meeting of the committee, held recently, under the presidency of Sir Thomas Morel, it was stated that up to the present £21,279 had been received. A piece of land of the value of £250, which adjoined the proposed site of the hospital, was presented by the Directors of the Taff Vale Railway Company.

Homes for Wounded Soldiers.—We learn that the authorities of the Porthcawl Rest, near Cardiff, have been approached by the

War Office as to the amount of accommodation which can be set aside for the sick and wounded on their return from Africa. The Cardiff Infirmary has offered 50 beds for the same purpose.

### CHELTENHAM.

OBITUARY.—On January 4th, Deputy Surgeon-General David Cullen, A.M.D., passed away peacefully at his residence, Halsey House, Cheltenham, in the sixty-seventh year of his age, after seven weeks' illness. Dr. David Cullen graduated M.D. Edin. and L.R.C.S. Edin. in 1854, and served in the Army Medical Department from 1854 to 1869. He served in the Crimea from 1854 to 1856 and in the Indian Mutiny from 1857 to 1859. Later he was Surgeon-Major to the 71st Highland Light Infantry, and was Registrar to the Royal Victoria Hospital, Netley, from 1875 to 1880, after which he retired on a pension. Dr. Cullen was awarded medals for his services in the Crimea, in Turkey, and in the Indian Mutiny, with clasps for Sebastopol and Lucknow. For the last 18 years Dr. Cullen had lived in Cheltenham.

### EXETER.

DEVON QUEEN VICTORIA COMMEMORATION FUND.—At a meeting of the Council of the Fund, held at Exeter on December 1st, 1899, under the presidency of the Earl of Morley, it was stated that about £6,450 had now been raised. The income of this amounts to £270, which will be devoted to various hospitals and infirmaries in the county of Devon.

### MALMESBURY.

PUBLIC HEALTH.—At a meeting of the Malmesbury Town Council, held on December 12th, 1899, the water-supply of the town came under discussion. The Medical Officer of Health (Mr. C. W. Pitt), who reported two fatal cases of typhoid fever, stated that the water was deleterious to health, and the county medical officer also said that unless the water was previously boiled it was unsafe for drinking purposes. After a considerable discussion it was decided to approach the company with a view to purchase of the waterworks by the Council.

### MERTHYR.

OBITUARY.—We have to record the death on January 20th, at the advanced age of 84 years, of Mr. Thomas Jones Dyke, who had held the position of medical officer of health of the town of Merthyr for over 36 years. He took his L.S.A. in 1837, and his M.R.C.S. in the year following. In 1866 he was elected a Fellow of the Royal College of Surgeons.

### PENZANCE.

WEST CORNWALL INFIRMARY AND DISPENSARY.—The annual meeting of this institution was held on January 17th, under the presidency of the Rev. Prebendary Hedgeland. The report stated that during 1899 there were 165 in-patients admitted, against 176 in 1898, and that 2,171 out-patients had been treated, as compared with 1991 in 1898. The financial statement showed that the total receipts amounted to £946, while the expenditure was £898.

### PLYMOUTH.

SOUTH DEVON AND EAST CORNWALL HOSPITAL.—The sixtieth annual meeting was held on February 6th, under the presidency of Sir Massey Lopes. The medical report stated that 1,244 in-patients had been admitted during 1899, being a decrease of 69 as compared with the previous year. 2,650 out-patients were attended, an increase of 314 as compared with 1898. 253 patients were sent to the Pearn Convalescent Home. The financial statement showed that the total expenditure amounted to £6,821, and there was an unfavourable balance of £802, but the balance from the bazaar held in 1898 enabled the committee to clear this deficit. Dr. R. H. Clay and Dr. L. E. Fox have been appointed Physicians. Mr. R. H. Lacy, Mr. C. Whipple, and Mr. W. Woollcombe have been appointed Surgeons. Mr. G. F. Aldous, Mr. C. E. R. Rendle, and Dr. H. W. Webber have been appointed Assistant Surgeons. Dr. F. Bushnell has been appointed Pathologist. Mr. G. R. Brittain has been appointed Surgeon-Dentist. Mr. C. H. Whiteford has been appointed Anæsthetist.

### SHEPTON MALLET.

District Hospital.—The annual meeting of this institution was held recently, under the presidency of Sir R. H. Paget. The medical report showed that 132 in-patients had been treated, against 124 in 1898. There were also 116 out-patients and 55 casualties treated, both being in excess of last year. The financial statement showed an increase under all heads, and the balance was satisfactory.

### SWINDON.

Victoria Hospital.—The annual meeting of this institution was held recently, under the presidency of Captain F. Goddard. The report showed that 225 patients had been admitted during 1899, and that there remained a favourable balance of £146. The chairman announced that he had given the land, which was said to be worth £300, for the enlargement of the hospital grounds. Captain Goddard had previously also given the site upon which the hospital stands.

### TAUNTON.

Taunton and Somerset Hospital.—At the annual meeting of this institution, held recently, the medical report stated that 777 in-patients had been admitted. The financial statement showed that there was a deficit on the year of £1,777, which had arisen partly through exceptional expenditure on the laundry and re-flooring the wards, and also to the fact that the amount received from donations and bequests had fallen off considerably. The fund raised in memory of the late Colonel Chard, V.C., had been increased to £909. It was decided to sell stock of the value of £1,000 to meet the deficiency in the accounts. The Committee decided to offer ten beds for the use of soldiers wounded in South Africa.

### TIVERTON.

Infirmary and Dispensary.—The annual meeting of this institution was held recently, under the presidency of Sir John Heathcote-Amory. The report showed that 1,293 patients had been treated. The Victoria wards, opened in August last, were now used by patients.

### TORQUAY.

Western Hospital for Consumption.—Mr. J. H. Gough has been appointed Honorary Physician *vice* Dr. J. R. Lombe, appointed Consulting Physician.

### TRURO.

Public Health.—The Corporation have adopted the aerobic bacterial oxidising process of the International Purification Syndicate for the treatment of the sewage of the town.

